# Identification of Major Phenolic Compounds from *Nephelium lappaceum* L. and Their Antioxidant Activities

**DOI:** 10.3390/molecules15031453

**Published:** 2010-03-09

**Authors:** Nont Thitilertdecha, Aphiwat Teerawutgulrag, Jeremy D. Kilburn, Nuansri Rakariyatham

**Affiliations:** 1 Division of Biotechnology, Graduate School, Chiang Mai University, Chiang Mai, 50200, Thailand; 2 Department of Chemistry, Faculty of Science, Chiang Mai University, Chiang Mai, 50200, Thailand; 3 School of Chemistry, University of Southampton, Southampton, SO17 1BJ, United Kingdom; E-Mail: jdk1@soton.ac.uk (J.D.K.)

**Keywords:** *Nephelium lappaceum*, antioxidant, geraniin, corilagin, ellagic acid, phenolic compounds

## Abstract

*Nephelium lappaceum* is a tropical fruit whose peel possesses antioxidant properties. Experiments on the isolation and identification of the active constituents were conducted, and on their antioxidant activity using a lipid peroxidation inhibition assay. The methanolic extract of *N. lappaceum* peels exhibited strong antioxidant properties. Sephadex LH-20 chromatography was utilized in the isolation of each constituent and the antioxidant properties of each was studied. The isolated compounds were identified as ellagic acid (EA) (**1**), corilagin (**2**) and geraniin (**3**). These compounds accounted for 69.3% of methanolic extract, with geraniin (56.8%) as the major component, and exhibited much greater antioxidant activities than BHT in both lipid peroxidation (77-186 fold) and DPPH^•^ (42-87 fold) assays. The results suggest that the isolated ellagitannins, as the principal components of rambutan peels, could be further utilized as both a medicine and in the food industry.

## 1. Introduction

**Figure 1:**
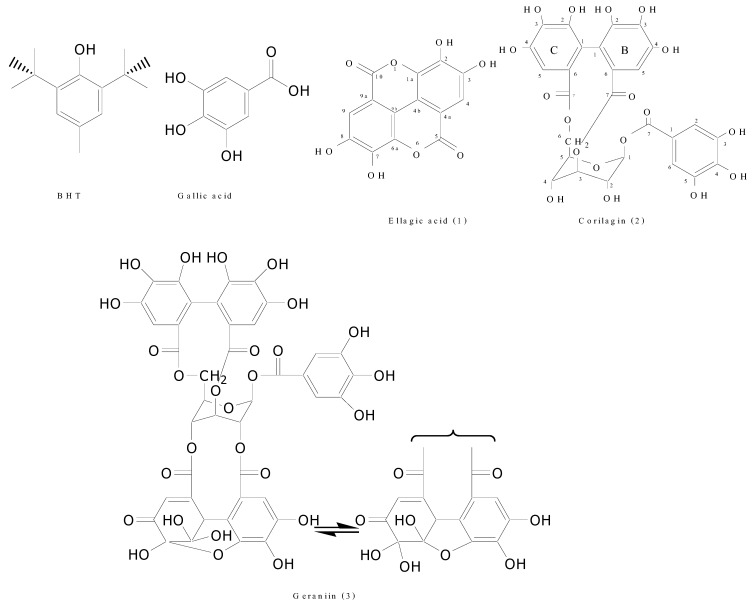
Chemical structure of BHT, gallic acid and three isolated phenolic components from *Nephelium lappaceum* L. peels: ellagic acid (**1**), corilagin (**2**) and geraniin (**3**) [3,9,10,11].

Free radicals and reactive oxygen species (ROS) are considered to be harmful to human health and play an important causative role in disease initiation [1,2]. Recently, the study of the isolation of natural antioxidants from plant sources has increased because synthetic antioxidants, such as BHT (butylated hydroxytoluene; Figure 1), are suspected of being responsible for liver damage and carcinogenesis [3]. Plants contain a large variety of substances possessing antioxidant activity, such as vitamin C, vitamin E, carotenes, xanthophylls, tannins and phenolics [4,5].

Vast quantities of agricultural-food waste are produced annually worldwide. In addition the disposal of agricultural-food waste can have a serious environmental impact. Nowadays, numerous investigations on waste reutilization have been aimed at evaluation of the waste materials in possible value-added applications. Agricultural waste, such as seeds and peel of grapes and/or pomegranate has been evaluated as a rich source of natural antioxidants [6,7,8]. 

*Nephelium lappaceum* L., commonly known as rambutan and belonging to the Sapindaceae, is an attractive tropical fruit widely distributed in South-East Asia, especially in the eastern and southern regions of Thailand. The approximate annual harvest quantity of rambutan is half a million tons. This fruit is consumed fresh, canned, or processed and its consumption results in the production of vast amounts of waste from seeds and peels of the fruit. In our previous study on rambutan waste, the antioxidant and phenolic content of its peel and seeds were evaluated [12]. Based on these studies we concluded that the seeds would not provide a viable source of antioxidants, but the peel waste has significant potential due to its powerful antioxidant properties and the large amounts of peel-materials being generated. 

As far as our literature survey could ascertain, no studies on the identification of phenolic compounds of the plant in the genus *Nephelium* have been previously published. This work is aimed at further examining the activity-guided fractionation of the components responsible for the high antioxidant properties in the methanolic extract from lyophilized *N. lappaceum* peel. Three individual compounds were identified from the methanolic extract and quantified. The structures of these compounds were identified by spectroscopic techniques and antioxidant analyses based on lipid peroxidation inhibition and DPPH^•^ scavenging assays performed.

## 2. Results and Discussion

### 2.1. Isolation and identification of bioactive compounds from N. lappaceum peel

**Figure 2:**
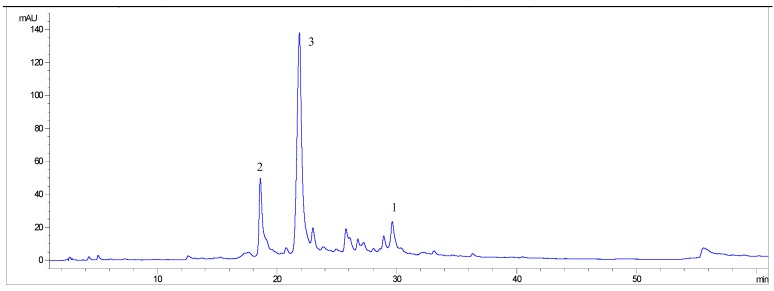
HPLC chromatogram of methanolic extract of *Nephelium lappaceum* L. peels. 1; ellagic acid, 2; corilagin, and 3; geraniin were detected at 280 nm.

The methanolic extract of *N. lappaceum* peel contained high amounts of phenolic compounds (542 mg/g dry extract) and exhibited obvious antioxidant activities (IC_50_ = 0.46 µg/mL on lipid peroxidation). This fraction was further purified using Sephadex LH-20 column chromatography, resulting in three main subfractions. Subfractions I, II and III were positive in lipid peroxidation inhibition and DPPH^•^ scavenging assays. These three subfractions were further analyzed by HPLC which gave a single peak (data not shown) in each case, corresponding to peaks 1, 2 and 3, respectively, of a typical HPLC profile of the methanolic extract (Figure 2). 

**Figure 3:**
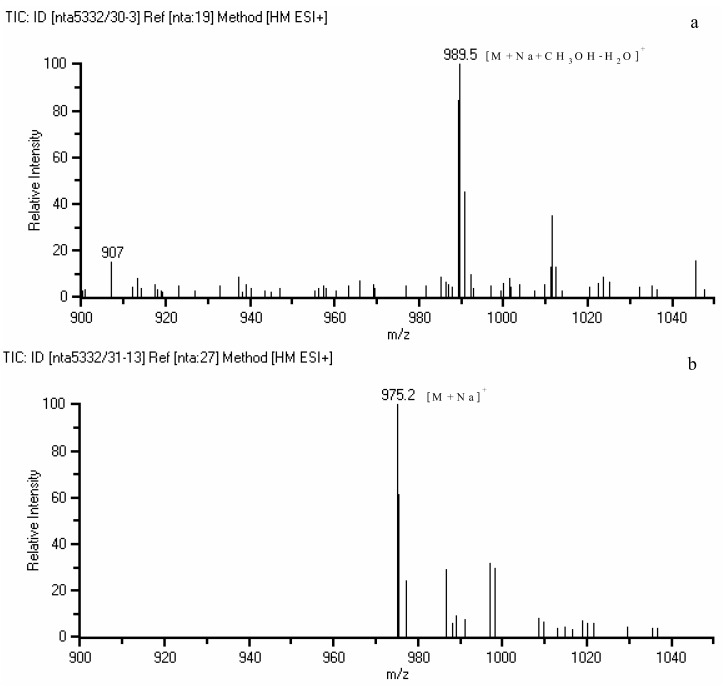
Mass spectra of geraniin dissolved in methanol (a) and dissolved in water after methanol has been removed (b).

**Figure 4:**
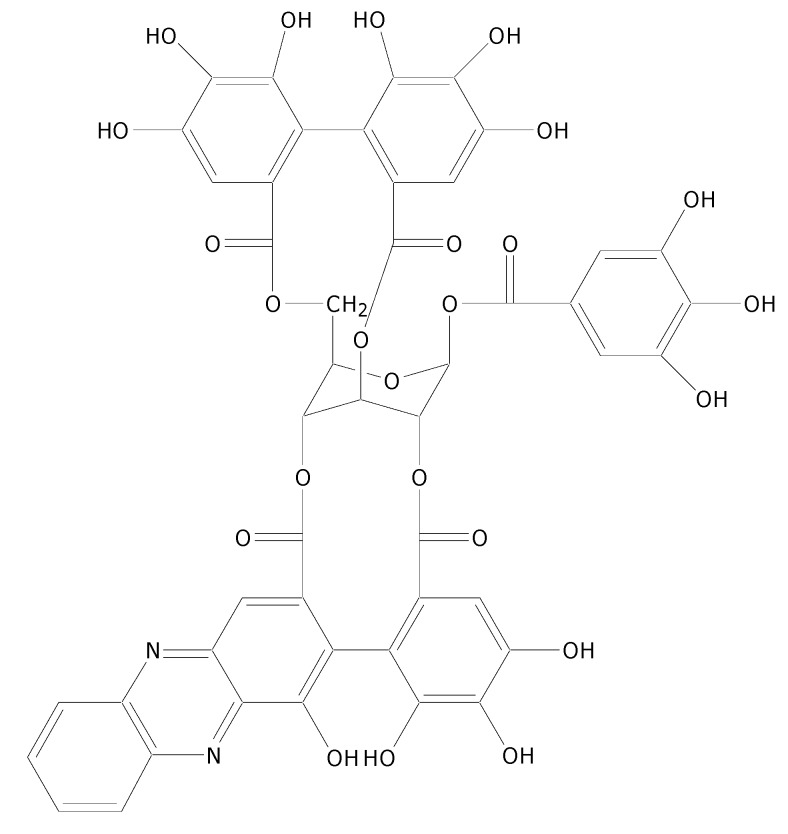
Chemical structure of phenazine-geraniin derivative [13].

The identification of the purified compounds was achieved by spectroscopic techniques and the spectroscopic data was directly compared with previously published data. The compound **1**, **2** and **3** were identified as ellagic acid, corilagin and geraniin, respectively. In the geraniin identification (compound **3**), the observation of methine carbon resonance in upfield regions (*δ* 46.19 and 51.97) of the ^13^C spectrum accompanied with hemiacetal carbon resonance (*δ* 92.38 and 96.21) and low field carbonyl carbon signals (*δ*191.71 and 194.43) indicated the presence of dehydrohexahydroxydiphenoyl (DHHDP) structure. The DHHDP moiety leads to the observation of complex spectra due to the doubling of NMR resonances because of its equilibrium mixture of five- and six membered hemiacetal rings [11]. The use of methanol in the extraction can lead to the formation of a methoxy hemiacetal instead of the geminal diol for both five- and six membered hemiacetal rings in geraniin. Thus a MS peak for [M+Na+CH_3_OH−H_2_O]^+^ (*m/z* value of 989) (Figure 3a) was observed instead of [M+Na]^+^ (*m/z* value of 975) but this formation is reversible to give back geraniin after methanol has been removed (Figure 3b). The problems associated with the multiplicity of NMR signals of DHHDP moiety were resolved by reaction with *o*-phenylenediamine to form the known [13] geraniin-phenazine derivative (Figure 4) which destroyed the hemiacetal ring. 

### 2.2. Quantification of bioactive identified components

The identified phenolic compounds in methanol extract of *N. lappaceum* peel were quantified using HPLC. As illustrated in Figure 2, the methanolic fraction of *N. lappaceum* showed EA (*t*_R_ 29.610 min), corilagin (*t*_R_ 18.597 min), and geraniin (*t*_R_ 21.856 min). For 1 g of methanolic extract, 53.5 mg of EA, 71.9 mg of corilagin, and 568.0 mg of geraniin were obtained. The total amount of the three identified compounds in this fraction was 693.4 mg/g extract. The HHDP residue of ellagitannins, including geraniin, is derived from penta-*O*-galloyl-*β*-D-glucose [14]. Geraniin when exposed to acids/bases, boiling water and *in vivo* are hydrolyzed to give corilagin, gallic acid and hexahydroxydiphenic acid, the latter have a high tendency to spontaneously undergo lactonization to afford EA (Figure 1) [15,16]. The low amount of EA and corilagin found in the methanolic extract may occur from the hydrolysis of geraniin during the development of *N. lappaceum* fruit or during the extraction process. However, gallic acid was not found in the current study (data not shown).

This is the first report on the isolation of EA, corilagin and geraniin from *N. lappaceum*. These identified compounds have been previously isolated from the other plants and have been reported to exhibit various antiviral [17,18], anti-inflammatory [19], apoptotic, cytotoxic [20,21], cytoprotective, antimicrobial and antioxidant [22,23] properties. The potent semicarbazide-sensitive amine oxidase and angiotensin I converting enzyme inhibitory activities, and hypertensive effects on spontaneously hypertensive rats (SHRs) of geraniin have also been reported. [24]. Importantly, there was not any toxicity to peripheral blood mononuclear cells (PBMC) and normal mouse fibroblast cells from rambutan-peel extract [25,26]. This suggests that *N. lappaceum* peels can be considered as a potential ellagitannin source.

### 2.3. Antioxidant activity

Lipid peroxidation plays an important role in causing oxidative stress in biological systems and several toxic byproducts from the peroxidation can disrupt biomolecule biochemical processes [27]. In the presence of antioxidants, the rate of lipid peroxidation can be reduced. The antioxidant activities of the three isolated phenolic compounds were determined by lipid peroxidation based on the ferric thiocynate method and compared with antioxidant standards (gallic acid and BHT). The lipid peroxidation inhibition increased along with their increased concentrations. The antioxidant properties are ranked as follows: geraniin (0.38 µM) > corilagin (0.70 µM) > EA (0.94 µM) > gallic acid (1.70 µM) > BHT (71.29 µM) (Table 1). Although EA activity was lower than those of all isolated phenolic compounds, EA was also reported to exhibit high lipid peroxidation inhibition induced by γ-radiation at low concentrations [28].

**Table 1:** The 50% inhibition concentration for lipid peroxidation inhibition and DPPH^•^ scavenging assays of ellagic acid, corilagin, and geraniin isolated from *Nephelium lappaceum* L. peels and comparison with reference antioxidant.

Compounds	Lipid peroxidation inhibition capacity		DPPH^•^ scavenging activity	
	IC_50_ (µM)	Mol BHT/mol phenolics	IC_50_ (µM)	Mol BHT/mol phenolics
Geraniin	0.38 ± 0.01 ^a^	186 ± 3.00	0.79 ± 0.05 ^a^	87.1 ± 5.76
Corilagin	0.70 ± 0.03 ^b^	103 ± 3.72	1.42 ± 0.10 ^b^	48.6 ± 3.44
Ellagic acid	0.94 ± 0.10 ^c^	76.9 ± 7.63	1.64 ± 0.15 ^b^	42.4 ± 3.85
Gallic acid	1.70 ± 0.13 ^d^	42.2 ± 3.10	2.49 ± 0.11 ^c^	27.7 ± 1.15
BHT	71.3 ± 0.86	-	68.8 ± 2.10	-

Values are expressed as means ± S.D. of three separate experiments; ^a^^-d^ Means the column followed by different letters are significantly different (P < 0.05).

The radical scavenging assay was based on the DPPH^•^ method which is one of the methods regularly used for measuring antioxidant potential by determining the free radical inhibitory ability of various antioxidants. Substances which undergo this reaction could be considered as antioxidants. Phenolic compounds have been described as those that exhibit powerful free radical inhibitor or scavenger properties [29]. The isolated compounds and the comparative standards (gallic acid and BHT) showed the dose-response radical scavenging activity. All isolated phenolic compounds exhibited greater scavenging activity than the comparative antioxidants (Table 1). The highest scavenging capacity was observed for geraniin (0.79 µM) followed by corilagin (1.42 µM), EA (1.64 µM), and gallic acid (2.49 µM), while BHT exhibited the lowest inhibition property (68.78 µM). 

Compared to the synthetic reference antioxidant (BHT), all isolated phenolic compounds and gallic acid possessed significantly greater activity, 42-186 fold and 28-87 fold, respectively, based on the lipid peroxidation inhibition and DPPH^•^ scavenging assays (Table 1). These results suggested that *N. lappaceum* can serve as a potent antioxidative agent. IC_50_ values of EA, corilagin, geraniin, and gallic acid on DPPH**^•^** scavenging assay have been reported by different authors (Table 2) [24,30,31,32,33]. All literature reports, including our own results, consistently reveal that increasing molecular weight of these compounds leads to enhanced antioxidant activity of the tested compounds. These are all powerful antioxidant compounds may be explained by the fact that they possess a great number of hydroxyl groups, and particularly many ortho-dihydroxy groups in the galloyl, HHDP and DHHDP units [23,31,34]. These three identified phenolic compounds have also been reported to possess the various strong ROS scavenging properties for superoxide anion (O_2_^·-^), hydroxyl radical (·OH) and peroxyl radical (ROO·) [22,24,28,35].

These results provide information on the potential use of *N. lappaceum* peels as well as the isolated phenolic compounds as antioxidative supplements. The large IC_50_ values of the identified compounds and their content level indicate that these are the major active components in the methanolic extract from *N. lappaceum*, with geraniin being the principal constituent.

From this point of view, our first study in phenolic isolation and identification of *N. lappaceum* peels can fulfill an important deficiency in the published research findings, can be a starting point for the study of the potential pharmacological properties of this plant species and the members of this genus and could also solve the problems of rambutan peel disposal. The research presented here is also an important introduction to *Nephelium lappaceum* as a potential food additive, as well as having cosmetic and pharmaceutical applications. Investigation of other biological activities, such as cytotoxicity against cancer cells and a study of protective effects on stress resistance in the model organism, may provide more evidence of the value to this fruit residue in the future.

**Table 2:** IC_50_ values of gallic acid, ellagic acid, corilagin and geraniin in DPPH radical scavenging assay from various literatures.

Reference	IC_50_ (µM)			
	gallic acid	ellagic acid	corilagin	geraniin
Zheng *et al*., 2009 [30]	4.71		3.17	
Latté *et al*., 2004 [31]	32.9		2.70	
Xu *et al*., 2007 [32]			27.4	18.7
Lin *et al*., 2008 [24]				1.27 (pH 7.9), 0.92 (pH 4.5)
Yokozawa *et al*., 1999 [33]	8.14	4.60	2.89	2.50
This work	2.49	1.64	1.42	0.79

## 3. Experimental

### 3.1. General

HPLC was performed in an Agilent 1100 UV-VIS high-performance liquid chromatography system (Agilent, Germany), equipped with a VertiSep column (4.6 × 150 mm, 5 µm, Vertical Chromatography, Thailand) and a gradient elution using water-MeCN (80%) from 100% water at 0 min up to 56:44 (v/v) at 50 min, then up to 100% MeCN after 55 min. Flow rate was 1.0 mL/min, 30 ºC. VWD (variable wavelength detector was performed at 280 nm [36]. ^1^H- (400 MHz) NMR and ^13^C- (100 MHz) NMR spectra were obtained on a Bruker DRX instrument (Bruker, Germany). High resolution-mass spectrometry (electrospray), HRMS (ESI) data were measured with QTOF mass spectrometer (Micromass, UK). IR spectra were recorded on a Nicolet 510 FT-IR spectrometer (Nicolet, USA). UV/Visible spectra were recorded on a Perkin-Elmer Lambda 25 UV-Vis spectrometer (Perkin Elmer instruments, USA). 1,1-Diphenyl-2-picrylhydracyl (DPPH^•^), gallic acid and BHT were obtained from Sigma-Aldrich (Steinheim, Germany). Sephadex LH-20 was purchased from Amersham Biosciences (Uppsala, Sweden). All other chemicals of analytical and HPLC grade were purchased from Merck (Darmstadt, Germany).

### 3.2. Extraction, isolation and identification of bioactive compounds from N. lappaceum peel

Extraction procedures were performed according to the method described previously [12]. The lyophilized peel powder of *N. lappaceum* L. (120 g) was extracted with ethyl ether (1 L) three times. The residue was then extracted with methanol (1 L) three times and finally with water (1 L) three times. After filtering each extract, the ethyl ether and methanol extracts were evaporated to absolute dryness (giving residues of 3.4 g and 30.1 g, respectively) and the aqueous extract was lyophilized (2.5 g). The bioactive methanolic fraction (10.0 g) was separated using Sephadex LH-20 column chromatography (4 × 55 cm) and eluted with methanol. Methanolic fractions (10 mL each) were collected and read at 280 nm. Based on the absorbance data, fractions were pooled to afford three subfractions which exhibited antioxidant property. These three subfractions, I, II, and III, were further analyzed for purity by HPLC to yield compound **1**, **2**, and **3**, respectively.

Compound **1** was obtained as a brown amorphous powder; UV λ_max_ (methanol) nm: 214, 216, 255, 366; IR (KBr) υ cm^-1^: 3,227, 1,701, 1,617, 1,446, 1,342, 1,200 cm^-1^; HR-ESI-MS *m/z*: 324.9960 [M+Na]^+^ (calcd. for C_14_H_6_O_8_Na^+^, 324.9960); ^1^H-NMR (CD_3_SOCD_3_): *δ* 7.40 (2H, s, H-4 and H-9); ^13^C-NMR (DMSO-*d6*): *δ* 106.6 (C-4b and C-9b), 109.9 (C-4 and C-9), 112.6 (C-4a and C-9a), 136.4 (C-1a and C-6a), 140.9 (C-2 and C-7), 148.4 (C-3 and C-8), 159.4 (C-5 and C-10). In comparison with authentic standard using HPLC, mass spectrum and ^1^H- and ^13^C-NMR spectra in the literature data [9,22], compound **1** was identified as ellagic acid (Figure 1). 

Compound **2** was obtained as a yellow amorphous powder; UV λ_max_ (methanol) nm: 220, 224, 275; IR (KBr) υ cm^-1^: 3,428, 1,712, 1,612, 1,352, 1,204 cm^-1^; HR-ESI-MS *m/z*: 657.0696 [M+Na]^+^ (calcd. for C_27_H_22_O_18_Na^+^, 657.0704); ^1^H-NMR (CD_3_COCD_3_): *δ* 7.13 (2H, s, H-2, 6 of galloyl proton), 6.85 (1H, s, H-5 of hexahydroxydiphenoyl portion (HHDP) ring C), 6.70 (1H, s, HHDP H-5 ring B), 6.38 (1H, s, glucose (Glc) H-1), 4.98 and 4.11 (2H, t, *J* = 11, dd, *J* = 2.8 and 8, CH_2_-Glc), 4.84 (1H, brs, Glc H-3), 4.53 (1H, t, *J* = 9.4, Glc H-5), 4.46 (1H, brs, Glc H-4), 4.06 (1H, brs, Glc H-2); ^13^C-NMR (CD_3_COCD_3_): *δ* 93.96 (Glc C-1), 75.51 (Glc C-5), 70.40 (Glc C-3), 68.77 (Glc C-2), 64.15 (Glc C-6), 62.06 (Glc C-4), 110.70 (Gal C-2,6), 120.72 (Gal C-1), 139.03 (Gal C-4), 145.71 (Gal C-3,5), 164.87 (Gal C=O), 107.97 (HHDP B-5), 109.77 (HHDP C-5), 115.76 (HHDP B-1), 116.27 (HHDP C-1), 125.40 (HHDP B-6), 125.66 (HHDP C-6), 136.53 (HHDP B-3), 137.00 (HHDP C-3), 144.63, 144.76, 144.79, 145.21 (HHDP B-2,4; C-2,4), 167.00 (HHDP C C=O), 168.37 (HHDP B C=O). From a detailed spectroscopic analysis and a comparison with the literature data [10,37], compound **2** was identified as corilagin (Figure 1).

Compound **3** was obtained as a yellow amorphous powder; UV λ_max_ (methanol) nm: 225, 279; IR (KBr) υ cm^-1^: 3,421, 1,719, 1,616, 1,448, 1,341, 1,210 cm^-1^; HR-ESI-MS *m/z*: 975.0695 [M+Na]^+^ (calcd. for C_41_H_28_O_27_Na^+^, 975.0716); ^1^H-NMR (CD_3_COCD_3_): *δ* 5.21 (methine), 7.31-6.57 (aromatic), 6.29-4.35 (sugar); ^13^C-NMR (CD_3_COCD_3_)*: δ* 46.19, 51.97, 62.35, 63.25, 63.66, 63.79, 65.90, 66.86, 69.93, 70.51, 72.60, 73.07, 73.30, 90.70, 91.77, 92.38, 96.21, 107.88, 108.04, 110.22, 110.52, 110.66, 110.86, 113.36, 115.21, 115.77, 119.57, 120.26, 124.60, 124.78, 125.66, 128.58, 136.42, 137.67, 139.68, 143.42, 144.51, 144.90, 144.96, 145.13, 145.23, 145.39, 145.73, 145.92, 154.57, 164.70, 164.81, 165.36, 165.56, 166.11, 168.17, 168.31, 191.71, and 194.43.

Phenazine-geraniin derivative was obtained as an orange powder; IR υ cm^-1^: 3,384, 1,718, 1,614, 1,508, 1,448, 1,312, 1,189 cm^-1^; LR-ESI-MS *m/z*: [M+H]^+^ 1007.3; ^1^H-NMR (CD_3_COCD_3_): 4.02 (1H, dd, *J* = 4.5 and 7.5), 4.78 (1H, dd, *J* = 4 and 8), 4.99 (1H, m), 5.44 (1H, d, *J* = 4), 5.53 (1H, d, *J* = 4.5), 5.64 (1H, d, *J* = 5.5), 6.17 (1H, d, *J* = 5.5), 6.70 (1H, s), 6.96 (1H, s), 6.97 (2H, s), 7.48 (1H, s), 7.99 (2H, m), 8.21 (1H, d, *J* = 8), 8.29 (1H, s), 8.32 (1H, d, *J* = 8); ^13^C-NMR (CD_3_COCD_3_): *δ* 65.10, 67.55, 68.57, 76.51, 76.74, 91.52, 108.26, 109.41, 109.99 (×2), 113.20, 114.69, 116.16, 116.34, 116.72, 119.84, 119.94, 120.33, 124.50, 125.35, 129.96, 130.44, 132.10, 132.21, 135.92, 136.14, 137.17, 139.05, 139.22, 139.45, 142.66, 143.06, 144.83, 144.92, 145.05, 145.18, 145.30, 145.69, 145.89 (×2), 152.10, 164.54, 165.98, 166.34, 167.66, 168.09. The identity of this fraction was achieved by comparison of its spectroscopic data, particularly complex ^13^C-NMR spectrum, with previous published data [13,38] and compound **3** was therefore confirmed as geraniin (Figure 1).

### 3.3. Phenazine derivative preparation

Compound **3** (50 mg) was dissolved in a solution of *o*-phenylenediamine (15 mg) in 20% acetic acid in ethanol (15 mL) and left to stand at room temperature overnight. Water (7.5 mL) was added and the resulting mixture was concentrated under reduced pressure. Addition of further water led to an orange coloured precipitate which was washed with water and dried under vacuum [13].

### 3.4. Analytical HPLC and quantification of bioactive components

Compounds **1**, **2**, and **3** were quantified using HPLC as described [36]. Each identified phenolic compound was used as a reference standard and dissolved in solvent to obtain five concentrations (0.010, 0.020, 0.050, 0.10, 0.25 mg/mL) for standard calibration curves. The amounts of each identified component in the methanolic extract of *N. lappaceum* were obtained on the basis of the quantity calculated from the standard calibration curve. The detection limit of each compound was determined at the concentration which S/N ratio was more than 3. 

### 3.5. Determination of antioxidant activities

#### 3.5.1. Linoleic peroxidation method

Determination of the antioxidant activity was based on the thiocyanate method described previously [39]. The sample was dissolved in methanol for stock solutions. Gallic acid and BHT were used as comparative standard. The sample solution at various concentrations in potassium phosphate buffer (2.5 mL, 0.04 mol/L, pH 7.0) was transferred to linoleic acid emulsion (2.5 mL) containing Tween-40 (0.28 g), linoleic acid (0.28 g) dissolved in potassium phosphate buffer (0.04 mol/L, pH 7.0). A control, containing linoleic acid emulsion (2.5 mL) and potassium phosphate buffer (2.5 mL, 0.04 mol/L, pH 7.0) was also prepared. The mixture was incubated at 37 ºC for 64 h. Aliquots (0.1 mL) were drawn from the incubation mixture at 8 h intervals and then mixed with 75% (v/v) ethanol (5.0 mL), 300 g/L ammonium thiocyanate (0.1 mL) and 20 mmol/L ferrous chloride (0.1 mL) in 0.96 mol/L HCl and allowed to stand at room temperature for 3 min. The absorbance was measured at 500 nm. The percent inhibition of lipid peroxidation was computed. The antioxidant activities were illustrated as IC_50_ values.

#### 3.5.2. *DPPH• scavenging activity*

The stable free radical-scavenging activity was determined by the 1,1-diphenyl-2-picryl-hydracyl (DPPH^•^) method [40]. A 0.1 mmol/L DPPH^•^ solution in methanol was prepared, and then an aliquot of this solution (1 mL) was mixed with sample (3 mL) at different concentrations. A control, containing DPPH^•^ solution (1 mL) and methanol (3 mL) was also prepared. The mixture was incubated at room temperature for 30 min and then the absorbance was measured at 517 nm. The ability to scavenge the DPPH^•^ was calculated as percent DPPH^•^ scavenging and concentration providing 50% inhibition (IC_50_) was calculated. Gallic acid and BHT were used for comparison.

### 3.6. Statistical analysis

All experimental results were expressed as means ± standard deviations. Analysis of variance was performed by ANOVA procedures. SPSS software was used for statistical calculations. The results with P < 0.05 were regarded to be statistically significant.

## 4. Conclusions

In summary the three main constituents in the methanolic extract of *N. lappaceum* peels were isolated based on an activity-guided fractionation method in one step column chromatography and were identified as EA, corilagin, and geraniin. The results obtained clearly demonstrate that the isolated constituents are responsible for the antioxidant activity of the methanolic extract of *N. lappaceum*. The combination of these identified compounds accounted for 69.3% of the methanol extract of rambutan peels. From our knowledge, this is the first report demonstrating isolation and identification of the compositions from *N. lappaceum* as possessing much greater antioxidant than the synthetic antioxidant (BHT). There is clear potential for the utilization of *N. lappaceum* peel as a food additive or a medicinal source and further investigation of its other biological activities lead us to believe that it may possesses additional value, as and need not simply be discarded.
